# From psychological imbalance to behavioral withdrawal: unraveling the impact of relative deprivation on organizational citizenship behavior in tourism enterprises

**DOI:** 10.3389/fpsyg.2025.1619960

**Published:** 2025-09-19

**Authors:** Shuangbin Han, Yuxiang Fan

**Affiliations:** School of Humanities and Social Sciences, Yancheng Institute of Technology, Yancheng, China

**Keywords:** relative deprivation, organizational citizenship behavior, organizational identification, attribution of responsibility, tourism enterprise

## Abstract

Relative deprivation (RD), an important antecedent of organizational citizenship behavior (OCB), remains underexplored in terms of its fundamental mechanisms. By integrating Social Identity Theory and Attribution Theory, this study investigates how RD affects OCB among 305 tourism employees, with organizational identification (OI) acting as a mediator and attribution of responsibility (AR) functioning as a moderator. Three key findings emerge: (1) RD significantly diminishes OCB; (2) OI partially mediates the negative relationship between RD and OCB; and (3) AR not only weakens the direct RD-OCB connection but also moderates the mediating role of OI. These findings advance the theoretical understanding of how negative psychological experiences influence OCB by clarifying the dual mechanisms of identification and attribution. Practically, this study provides actionable strategies for tourism organizations to improve employee behavior through fair frameworks, cultural empowerment, and attributional guidance.

## 1 Introduction

Amid global competition and digital transformation in the service industry, tourism enterprises face increasing market pressures and organizational changes, making employees’ organizational citizenship behavior (OCB) a crucial foundation for enhancing service efficiency and maintaining competitive differentiation (Cordeiro et al., 2024; Tuan et al., 2021). OCB, defined as voluntary actions that extend beyond formal job requirements (e.g., helping coworkers and organizational loyalty), is influenced by extrinsic incentives and employees’ intrinsic psychological states (Turnipseed, 2002). However, existing research has primarily focused on the positive role of psychological resources, such as psychological capital and thriving at work, in fostering OCB (Jamal et al., 2023; Liu et al., 2024; Luthans et al., 2007), while largely ignoring the detrimental effects and underlying mechanisms of negative psychological experiences, especially relative deprivation (RD). This theoretical oversight poses a risk of managerial neglect toward the latent threats arising from employees’ psychological imbalances, potentially undermining the organizational resilience.

RD is a psychological state arising from perceived discrepancies between individuals’ expectations and actual outcomes through social comparisons (Walker and Smith, 2002). This demands increased attention in tourism, an industry characterized by intense emotional labor. Employees in tourism enterprises often face stressors such as wage disparities, career stagnation, and customer conflicts (Choi et al., 2000; Costa et al., 2017; Lee and Madera, 2019), which may exacerbate RD and subsequently influence behavioral choices through cognitive and affective pathways (Smith et al., 2012). However, existing findings on the relationship between RD and OCB are controversial. While some scholars argue that RD leads to counterproductive outcomes, including withdrawal and deviance (Li et al., 2022; Schreurs et al., 2021), while others propose that RD may paradoxically inspire compensatory efforts under certain conditions (Karacay et al., 2023; Wang et al., 2023). The variability in these findings highlights the need for a more sophisticated theoretical framework capable of elucidating the intricate psychological mechanisms involved.

To address this complexity, this study integrates Social Identity Theory and Attribution Theory. Social Identity Theory offers a foundational perspective, suggesting that individuals derive a portion of their self-concept from their membership in social groups, including their organization (Ashforth and Mael, 1989). This sense of unity, referred to as organizational identification (OI), promotes prosocial behaviors such as OCB because supporting the organization becomes synonymous with supporting one’s self (Dai et al., 2022). When employees experience deprivation, their psychological connection to and identification with the organization are likely to be compromised. However, Social Identity Theory alone does not fully account for the significant variation in the impact of RD on OI and subsequent behaviors among individuals.

Attribution Theory provides a vital complementary perspective for understanding human behavior. This theory elucidates the process by which individuals interpret and assign causality to events (Weiner, 1985). It posits that our emotional and behavioral responses are influenced not by the event itself but by our attribution of its cause. For example, we may attribute the cause to internal factors, such as personal effort, or to external factors, such as managerial bias or systemic inequity (Zuo et al., 2023).

The integration of Social Identity Theory and Attribution Theory offers a more robust explanatory framework for comprehending the consequences of RD. We posit that an employee’s causal attribution for their experienced deprivation serves as a crucial psychological mechanism that influences the impact of RD on their OI. When employees attribute their deprivation to external and stable factors controlled by the organization, such as unfair policies or biased managers, they are more inclined to perceive their organization as an unjust outgroup. This external attribution can directly undermine their social identity, erode their OI, and consequently suppress their OCB as a form of behavioral withdrawal (Foster and Rusbult, 1999). Conversely, if employees attribute their deprivation to internal or unstable factors, such as their own temporary lack of skills or effort, the negative impact on their OI may be mitigated. This integrated perspective aids in explaining the inconsistent findings in the literature and provides a solid theoretical foundation for the moderated mediation model.

By synergizing these two theories, this study develops a comprehensive framework to investigate the psychological processes linking RD to OCB and address three specific questions: (1) Does RD negatively affect OCB in tourism enterprises? (2) Does OI mediate the relationship between RD and OCB? (3) Does AR moderate the strength of the RD-OI relationship and the mediating role of OI in the connection between RD and OCB? Theoretically, this study enhances our understanding of the complex pathways through which negative psychological states inhibit OCB, challenges the entrenched “positivity bias” in the OCB literature (Bolino et al., 2013), and elucidates the dynamic role of OI. Practically, it offers actionable insights for tourism enterprises to refine human resource strategies—for instance, by fostering OI to mitigate the detrimental effects of RD or guiding adaptive AR to recalibrate employees’ cognitive and behavioral responses.

## 2 Theoretical foundation and research hypotheses

### 2.1 Theoretical foundation

This study is grounded in the Evaluation-Emotion-Response (EER) theoretical framework (Bagozzi, 1992). Emerging from the confluence of cognitive psychology and affective science, the EER framework underscores a tripartite dynamic process through which individuals interpret and react to external stimuli: cognitive Evaluation, emotional Experience, and behavioral Response. Its fundamental premise asserts that “cognition triggers emotion, and emotion drives behavior.” This theoretical framework offers an integrated explanatory approach to reveal the mechanism by which RD affects OCB in the context of tourism enterprises.

#### 2.1.1 Relative deprivation

RD is a socio-psychological construct introduced by Stouffer et al. (1949) to describe perceptions of structural injustice resulting from discrepancies between expected entitlements and actual gains. This concept emphasizes that deprivation arises not from absolute resource scarcity but from cognitive and emotional imbalances developed through social comparisons with reference groups (Smith et al., 2012).

The mechanisms underlying RD can be interpreted using two theoretical frameworks. First, Social Comparison Theory posits that individuals assess their status through horizontal (interpersonal) or vertical (temporal self-referential) comparisons. Disadvantageous outcomes from these comparisons, such as perceived injustices in compensation or promotions among tourism employees, may trigger RD (Ohno et al., 2023). Second, Value Expectancy Theory suggests that RD emerges from discrepancies between normative expectations (e.g., fair rewards) and actual experiences, which structural factors such as organizational allocation systems or hierarchical barriers can often exacerbate (Walker and Smith, 2002). In tourism, seasonal demand fluctuations and unpredictable service interactions heighten employees’ sensitivity to unmet expectations, further amplifying their RD (Jolliffe and Farnsworth, 2003).

RD manifests in two dimensions: individual relative deprivation (IRD) and group relative deprivation (GRD) (Tropp and Wright, 1999). IRD refers to personal feelings of unfairness regarding compensation, career advancement, or working conditions, which are typically accompanied by frustration and linked to outcomes such as decreased organizational commitment and increased turnover intention (Yu et al., 2025). In contrast, GRD arises when members collectively perceive their group (e.g., department, team) as disadvantaged compared to others in terms of resources or status. This collective sense of deprivation can undermine team cohesion, escalate intra-organizational conflict, and trigger collective resistance behaviors (Guimond and Dubé-Simard, 1983; Power et al., 2020).

Critically, the IRD and GRD dynamically intersect. Group-level disadvantages can amplify individual deprivation, whereas the GRD affects individual behavioral choices through social identification (Smith et al., 2012). For instance, an employee facing IRD due to unfair compensation may experience increased organizational alienation if their department is institutionally marginalized, resulting in a ‘dual deprivation’ effect. These interactions highlight the complexity of RD in organizational contexts, especially in tourism enterprises, where role ambiguity and cross-team comparisons are prevalent.

#### 2.1.2 Organizational citizenship behavior

OCB refers to employees’ voluntary actions that go beyond formal job requirements, remain unrecognized by organizational reward systems, and collectively enhance the organization’s functionality (Organ, 1988). Its theoretical significance lies in demonstrating how employees’ spontaneous contributions implicitly drive organizational resilience and collaborative efficiency. OCB typically encompasses altruism, courtesy, sportsmanship, conscientiousness, and civic virtue (Lee and Shin, 2024). Altruism refers to employees’ voluntary assistance to colleagues in completing tasks; courtesy involves employees showing respect and politeness toward colleagues; sportsmanship indicates employees’ willingness to take on extra work burdens; conscientiousness reflects employees’ dedication and responsibility toward their work; and civic virtue pertains to employees’ identification with the organization’s goals and values (Podsakoff et al., 2000). Although not officially required, these behaviors foster positive work environments, strengthen team cohesion, and enhance organizational performance (Kao et al., 2023). For instance, altruistic actions, such as helping colleagues solve problems or sharing essential operational knowledge, improve team coordination efficiency. Similarly, polite behavior reduces interpersonal conflicts, promoting a harmonious workplace climate that supports sustained collaboration.

Multiple factors influence the emergence and display of OCB. At the individual level, personality traits such as conscientiousness, cognitive tendencies such as psychological ownership, and emotional states such as job embeddedness are significantly correlated with OCB engagement (Anitha et al., 2024). Employees with high self-efficacy are more likely to make role-expanding contributions. On an organizational level, transformational leadership enhances OCB by inspiring responsibility through inspirational motivation and individualized consideration (Lee et al., 2024), whereas supportive cultures reinforce their sustainability through psychological safety mechanisms (Sumardjo and Supriadi, 2023). When employees perceive organizational support, such as fair compensation and career development opportunities, they tend to reciprocate through increased OCB (Gupta et al., 2024; McManus et al., 2025). Additionally, Job-design characteristics further shape OCB dynamics. Task autonomy empowers employees to perceive the meaningfulness of discretionary contributions, whereas task interdependence fosters cooperative norms through socially embedded expectations (Grant and Parker, 2009). Transparent promotion frameworks in hospitality enterprises strengthen organizational trust and motivate employees to engage in OCB, such as voluntarily mentoring new hires (Golverdi et al., 2024).

Employees’ job characteristics within tourism enterprises—a quintessential service-intensive sector—exhibit high role extensibility and context dependence (Baum, 2018). Seasonal demand fluctuations, non-standardized client expectations, and dynamic operational environments frequently necessitate that employees transcend formal role boundaries (Teng, 2019). Typical OCB manifestations include assuming additional responsibilities during peak seasons, adapting to flexible scheduling demands, and proactively safeguarding the organization’s reputation of the organization. These behaviors constitute strategic resources for navigating competitive landscapes and achieving service differentiation (Organ, 1988), underscoring the managerial imperative to cultivate OCB in the tourism workforce.

#### 2.1.3 Organizational identification

OI, an extension of Social Identity Theory into organizational contexts, refers to the psychological process by which individuals integrate organizational characteristics, values, and goals into their self-concept through cognitive and emotional alignment (Ashforth and Mael, 1989). At its core, OI reflects employees’ perceived congruence between their self-definition (“who they are”) and the organizational identity (“what the organization represents”). This alignment manifests through the acceptance of organizational norms, internalization of collective objectives, and voluntary synchronization of personal interests with organizational goals (Bergami and Bagozzi, 2000).

OI encompasses three interrelated dimensions: cognitive, affective, and evaluative (Van Knippenberg and Sleebos, 2006). The cognitive dimension involves the rational recognition of organizational membership (e.g., “I am part of this company”). The affective dimension reflects emotional attachment to the organization (e.g., “I feel proud to work here”). In contrast, the evaluative dimension relates to self-worth assessments derived from an organization’s societal status (e.g., “The company’s reputation enhances my public image”) (Ashforth and Mael, 1989). The dynamic interaction among these dimensions influences the strength of identity integration and significantly shapes employees’ work attitudes and behavioral choices (Lee et al., 2015).

The formation and evolution of the OI are affected by various factors. Person-Organization Values act as a foundational antecedent. Employees show increased identification when they perceive an alignment between their personal values and organizational culture, such as innovation orientation or social responsibility (Tourky et al., 2023). Leadership behavior also plays a critical role in meaning-making and emotional mobilization in the workplace. For instance, transformational leaders nurture OI by articulating shared visions and empowering employees to view organizational missions as personal objectives. Conversely, ethical leaders strengthen OI by building trust in the organization’s legitimacy through equitable decision making (Walumbwa et al., 2008). Furthermore, perceived organizational support, such as access to career development resources and job autonomy, reinforces identification by addressing employees’ psychological needs for belongingness and esteem (Kurtessis et al., 2017).

Regarding outcomes, OI not only boosts individual job engagement and performance but also promotes organizational innovation by enabling cross-departmental collaboration and tacit knowledge sharing (Divya and Christopher, 2024; Riketta, 2005). In service-intensive industries, such as tourism, OI’s supportive role is especially significant. By reducing role stress and enhancing service resilience, OI directly impacts customer satisfaction and organizational competitiveness (Karatepe, 2013). For example, frontline employees with strong identification are more likely to demonstrate proactive service recovery behaviors during customer conflicts, thereby preserving the brand reputation.

#### 2.1.4 Attribution of responsibility

AR refers to a distinctive cognitive process through which individuals interpret or infer the causes of events or actions (Su et al., 2020). This cognitive process significantly influences subsequent emotional states, motivational intensity, and behavioral patterns (Tiamiyu et al., 2020). Individual attitudes and behaviors are shaped by two attribution styles: internal and external (Weiner, 1985). Internal attribution involves assigning causality to inherent personal traits, such as abilities, motivations, or levels of effort. In contrast, external attribution emphasizes contextual factors such as situational constraints, task difficulty and interpersonal influence (Zuo et al., 2023).

Empirical evidence suggests that cultural contexts profoundly shape attribution patterns (Hsu and Chen, 2019). For instance, Confucian values emphasize introspection and self-cultivation, which have ingrained internal attribution tendencies in the Chinese cultural psychology. Consequently, Chinese individuals often attribute adversity to internal factors (e.g., personal competence or diligence) rather than external circumstances. Notably, internal attribution tends to exhibit greater stability and evoke stronger emotional responses (Weiner, 1985), reflecting individuals’ proactivity and agency in navigating social phenomena and providing deeper insights into their psychological and behavioral mechanisms. Given this theoretical rationale, the current study examines internal attribution to clarify how it moderates the mechanism by which RD influences OCB through the internal psychological state of tourism employees.

### 2.2 Research framework and hypothesis

#### 2.2.1 Research framework

**Figure 1:**
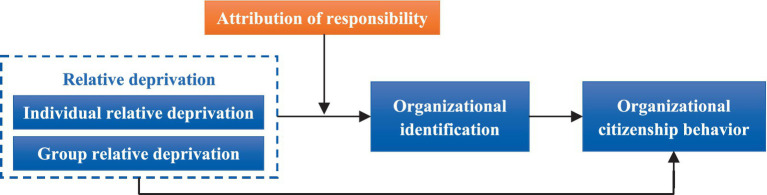
Research model.

This study constructs an integrated model (Figure 1) based on the Evaluation-Emotion-Response (EER) theoretical framework (Bagozzi, 1992) to examine how RD influences OCB among hospitality industry employees. This research systematically unveils the transmission pathway of “psychological deprivation → emotional identification → behavioral choice” and its boundary conditions. Within the hospitality context, employees’ cognitive appraisal of RD—such as perceived deserved-received discrepancy through horizontal (peer-based) or vertical (industry-standard-based) comparisons—may trigger negative affective experiences (e.g., organizational alienation). This subsequently suppresses employees’ voluntary adoption of extra-role behavioral responses (e.g., OCB) through emotion-driven mechanisms (Junça-Silva and Lopes, 2023). This causal chain aligns with the EER theory’s core logic wherein “cognition activates emotion, and emotion drives behavior.”

Expanding the applicability of the EER framework, the proposed model positions RD as the antecedent variable, OI as the mediator, and OCB as the outcome variable, incorporating AR as a key moderator. According to EER principles, RD, as a cognitive evaluation, weakens employees’ emotional connection to the OI, thereby indirectly inhibiting OCB (Bagozzi, 1992). For instance, employees who perceive RD through unfavorable compensation comparisons may reduce their discretionary contributions due to emotional detachment (Li et al., 2022). Additionally, AR—individuals’ causal interpretations of deprivation (e.g., self-related factors vs. organizational inequity)—moderates the strength of RD’s negative influence on OI. By integrating the EER framework with Attribution Theory, this study advances a moderated mediation model (Figure 1) that systematically unravels the “psychological deprivation → emotional identification → behavioral choice” pathway and its contextual boundaries.

#### 2.2.2 Relative deprivation and organizational citizenship behavior

IRD arises from negative psychological experiences in which individuals perceive themselves as disadvantaged through social comparisons with in-group or out-group members (Guimond and Dubé-Simard, 1983). These interpersonal comparisons significantly influence employees’ attitudes and behaviors. Psychological research shows that IRD generates discontent and anger (Smith et al., 2012), intensifies perceptions of organizational injustice (Melkonian et al., 2011), and reduces cooperative intentions (Organ, 1988). Specifically, employees may decrease their OCB as a low-risk response to perceived inequity, indirectly voicing their dissent (Bolino et al., 2013). Additionally, upward social comparisons, which involve comparing oneself to higher-status peers, are crucial antecedents of IRD (Marescaux et al., 2021). When employees perceive disparities in resources or status compared to their colleagues, they may resort to maladaptive coping strategies, such as avoidance or downward comparisons, to relieve cognitive dissonance. However, these strategies contribute to disengagement from organizational objectives and diminish proactive participation in OCB. From an organizational behavior standpoint, IRD can lead employees to withhold knowledge as proprietary assets (Chi and Han, 2008) or even trigger counterproductive workplace behaviors to vent feelings of deprivation (Schreurs et al., 2021).

GRD emerges from intergroup comparisons, wherein in-group members perceive systemic disadvantages compared to external groups (Smith and Ortiz, 2002). GRD hinders OCB through three mechanisms. First, it undermines group cohesion and trust foundations, when members question the fairness of shared goals or leadership decisions, their willingness to collaborate significantly decreases (van Zomeren et al., 2008). Second, it worsens negative work attitudes: groups experiencing chronic disadvantage develop collective frustration, leading to diminished work engagement and innovation capacity (Pettigrew et al., 2008). Third, it prompts aggressive collective actions (Su et al., 2023), wherein employees may engage in extreme measures, such as strikes, to secure collective interest restitution (Wright et al., 1990), which are inherently incompatible with the altruistic nature of OCB (Podsakoff et al., 2000). In tourism enterprises, these mechanisms may be amplified by industry-specific dynamics, such as seasonality. For instance, cross-departmental disparities in performance-based rewards (e.g., between tour guides and hotel staff) increase the frequency and intensity of intergroup comparisons. The high emotional labor demands of tourism may exacerbate the GRD’s detrimental effects. When employees perceive ongoing inequities disadvantaging their group, emotional exhaustion reduces their willingness to take on discretionary responsibilities (Cropanzano et al., 2003). Based on these insights, this study proposes the following hypotheses:

*H1a:* IRD negatively influences OCB.

*H1b*: GRD negatively influences OCB.

#### 2.2.3 The mediating role of organizational identification

The mediating role of OI in enhancing OCB has received considerable theoretical support. Grounded in Social Identity Theory, employees with a strong OI align their self-concept with organizational goals (Ashforth and Mael, 1989), engaging in identity-consistent behaviors such as role expansion and maintaining collective interests. These identification-driven actions encompass improved efficiency and extra-role contributions (e.g., peer assistance and innovative suggestions) and strategic investments in organizational sustainability (Riketta, 2005). For instance, Dukerich et al. (2002) demonstrated that employees with high OI disproportionately defend organizational reputation, even when these efforts exceed formal job requirements. Similarly, Kurtessis et al. (2017) revealed that heightened OI enhances perceived organizational support and motivates OCB through social exchange mechanisms.

This relationship functions through two pathways: psychological attachment and social exchanges. Psychologically, OI fosters affective commitment (Meyer and Allen, 1991), creating emotional bonds that lead employees to internalize organizational interests as extensions of their self-concept. Consequently, individuals spontaneously enact OCB to uphold shared values (Van Knippenberg and Sleebos, 2006). From a social exchange perspective, employees reciprocate perceived organizational support (e.g., fair compensation and developmental opportunities) by engaging in OCB, guided by norms of reciprocity (Cropanzano and Mitchell, 2005). Eisenberger et al. (2001) suggested that when OI reinforces employees’ self-perception as organizational agents, they are more likely to view OCB as a reciprocal contribution to organizational investment rather than an extraneous burden. This dynamic is particularly noticeable in the service industry. Teng et al. (2020) found that hospitality employees with strong OI define their professional identity through organizational attributes and exhibit behaviors aligned with institutional objectives. Based on the above analysis, this study proposes the following hypothesis:

*H2*: OI has a positive influence on OCB.

As a psychological process through which individuals integrate their self-concept with organizational attributes (Cheney, 1983), OI is fundamentally an identity construction achieved via social categorization. Social Identity Theory posits that employees derive group belongingness and self-enhancement through OI, with its intensity contingent upon perceived organizational distinctiveness and appeal (Ashforth and Mael, 1989). When individuals recognize significant resource disparities (e.g., compensation and promotion opportunities) relative to reference groups through upward comparisons, the resultant RD triggers cognitive dissonance. This dissonance diminishes alignment with organizational values (Kreiner and Ashforth, 2004) and fosters emotional detachment, manifesting as indifference toward organizational goals and withdrawal from extra-role contributions (Van Knippenberg and Sleebos, 2006).

Existing research indicates that RD weakens employees’ organizational embeddedness, thereby indirectly affecting their prosocial behavior (Li et al., 2022). Specifically, when employees perceive RD, they tend to view the organization as an “out-group,” which reduces their intrinsic motivation to engage in OCB (Osborne et al., 2015). In this process, OI serves as a mediating variable, bridging the psychological path between RD and OCB: high deprivation erodes identification, thereby reducing employees’ voluntary contributions (such as helping colleagues or proactive innovation). Empirical evidence shows that this mediating effect is particularly pronounced in high mobility industries (Dai et al., 2016). Furthermore, IRD often influences OCB through personal-level attenuation of identification, whereas GRD may amplify collective-level identification deficits, leading to a decline in overall team OCB (Smith and Pettigrew, 2015). In tourism enterprises, seasonal demand fluctuations and the high mobility of frontline employees exacerbate this effect, making employees more prone to amplifying deprivation through social comparison, thus weakening loyalty and commitment to the organization (Pan and Yang, 2023). Therefore, OI is not only a direct outcome of RD but also a key mediating mechanism for the withdrawal of the OCB. Based on this rationale, the following hypotheses are proposed:

*H3a*: OI mediates the relationship between IRD and OCB.

*H3b*: OI mediates the relationship between GRD and OCB.

#### 2.2.4 The moderating role of responsibility attribution

The negative impact of RD on OI does not follow a linear transmission; its intensity and direction are systematically moderated by individuals’ attributional styles (Weiner, 1985). Grounded in attribution theory, when employees attribute RD experiences to internal controllable factors (e.g., personal effort and skill development), their cognitive appraisal systems activate self-regulatory mechanisms (Carver and Scheier, 1998). This tendency to seek internal attributions motivates individuals to adopt problem-solving strategies, such as participating in skill enhancement programs (e.g., service quality initiatives in tourism firms) or improving performance metrics (e.g., client relationship management competencies) (Dweck, 2013). Such adaptive responses mitigate negative emotions while preserving psychological attachment to the organization itself (Li et al., 2022). For example, hotel employees attributing promotion delays to inadequate language proficiency might proactively enroll in training courses instead of questioning organizational fairness, thereby maintaining their OI through cognitive reframing.

Conversely, external attributions (e.g., inequitable resource distribution and managerial favoritism) trigger negative emotional cascades (Li et al., 2022). This attributional pattern generates two sequential outcomes: First, it reinforces persistent RD perceptions through intensified social comparisons (Kim et al., 2018). Tour guide teams, for instance, might develop prolonged grievances when they compare their welfare packages with those of hotel departments. Second, it accelerates organizational alienation behaviors, such as restricted knowledge sharing and avoidance of cross-functional collaboration, thereby hastening OI erosion (Chaudhary et al., 2024; Eberly et al., 2011). Empirical studies have shown that AR moderates the relationship between adverse experiences and their consequences (Bugental, 1987). Strong internal attribution tendencies weaken the negative association between RD and positive affective states (Li et al., 2022). Consequently, this study proposes the following hypothesis:

*H4a*: AR moderates the negative relationship between IRD and OI.

*H4b*: AR moderates the negative relationship between GRD and OI.

Building on previous theoretical insights, RD (including IRD and GRD) influences OCB through the mediating mechanism of OI, with AR moderating the relationship between RD and OI. Importantly, when employees attribute RD to self-referential factors, such as personal effort, the negative impact of RD on OI diminishes, thereby reducing its suppressive effect on OCB. This suggests that AR regulates the strength of the indirect pathway by which RD impacts OCB via OI. Therefore, this study proposes the following moderated mediation hypothesis:

*H5a*: AR moderates the mediating role of OI in the relationship between IRD and OCB.

*H5b*: AR moderates the mediating role of OI in the relationship between the GRD and OCB.

## 3 Research methodology

### 3.1 Variable measurement

The measurement scales used in this study were adapted from validated instruments found in the existing literature, with contextual modifications to ensure theoretical alignment and methodological rigor of the study. IRD was operationalized using the three-item scale developed by Tropp and Wright (1999). GRD was assessed using a refined version of Liang et al.’s (2019) 14-item scale, retaining four items after a systematic screening process to enhance contextual relevance. OI was measured using Mael and Ashforth’s (1992) six-item scale, and OCB was evaluated using a condensed version of Podsakoff et al.’s (1990) instrument, which comprises four context-specific items. AR was measured using a four-item scale validated by Zhang et al. (2020). All scales underwent pilot testing (*n* = 98) to verify their reliability and remove any ambiguous phrasing. The finalized instruments utilized a 7-point Likert response format ranging from 1 (“strongly disagree”) to 7 (“strongly agree”).

### 3.2 Sample and data

Data were collected through an online survey of employees from various tourism enterprises, including travel agencies, hotels, scenic areas and tourist shops. The survey was distributed via the Questionnaire Star platform between July 1 and October 30, 2024.

A snowball sampling strategy was used for the recruitment of participants. The questionnaire link, embedded with a QR code, was disseminated through social media and organizational communications. This method facilitated chain-referral sampling by utilizing existing professional networks to improve response rates, while ensuring sector relevance.

The initial data collection yielded 338 responses. After rigorous quality control procedures to eliminate invalid entries (e.g., duplicate responses and patterned selections), 305 questionnaires were retained for analysis, resulting in a valid response rate of 90.24%. Female participants comprised 53.11% of the respondents, while males accounted for 46.89% of the respondents. Age distribution showed 4.92% in the 18–20 age group, 44.92% aged 21–30, 34.10% aged 31–40, and 16.07% over 40. Regarding educational attainment, 35.74% had high school/vocational diplomas, 32.46% held college/bachelor’s degrees, and 29.84% reported junior high school education or below. The sector representation comprised hotel employees (47.54%), scenic area staff (24.59%), and tourist shop employees (12.79%).

The sample size of 305 respondents was determined to be adequate via *a priori* power analysis using G*Power 3.1 software (Faul et al., 2007), assuming a medium effect size (f^2^ = 0.15), an alpha value of 0.05, and a power of 0.95 for hierarchical regression analysis. This ensures sufficient statistical power to detect relationships in the tourism enterprise context.

## 4 Analysis and results

### 4.1 Common method bias test

Given the cross-sectional nature of the data collection, where respondents self-reported all variables simultaneously, we thoroughly assessed potential common method bias (CMB) to ensure methodological validity of the study. Following established protocols (Podsakoff et al., 2003), we conducted Harman’s single-factor test. All observed variables in the research model underwent exploratory factor analysis (EFA) without rotation. The results showed that the first extracted factor accounted for 33.19% of the total variance, below the 40% critical threshold widely cited in methodological literature. This outcome confirms that the common method bias did not significantly distort the study’s findings.

### 4.2 Reliability and validity evaluation

Scale reliability was assessed using Cronbach’s alpha coefficients and composite reliability (CR) values. As shown in Table 1, both Cronbach’s alpha values and CR scores for all constructs surpassed the recommended threshold of 0.7, indicating satisfactory internal consistency.

**Table 1:** Results of the measurement model.

Construct	Item	Std. Loading	t-statistic	CR	AVE	Cronbach’s *α*
Individual relative deprivation	IRD1	0.782	—	0.820	0.603	0.819
	IRD2	0.757	12.16			
	IRD3	0.791	12.48			
Group relative deprivation	GRD1	0.798	—	0.889	0.666	0.887
	GRD2	0.816	15.35			
	GRD3	0.788	14.72			
	GRD4	0.861	16.31			
Organizational identification	OI1	0.849	—	0.928	0.681	0.926
	OI2	0.779	16.33			
	OI3	0.828	17.99			
	OI4	0.797	16.92			
	OI5	0.822	17.76			
	OI6	0.874	19.68			
Organizational citizenship behavior	OCB1	0.728	—	0.857	0.601	0.857
	OCB2	0.773	12.52			
	OCB3	0.798	12.88			
	OCB4	0.800	12.90			
Attribution of responsibility	AR1	0.754	—	0.854	0.595	0.852
	AR2	0.727	12.04			
	AR3	0.780	12.88			
	AR4	0.822	13.44			

Confirmatory factor analysis (CFA) was conducted to evaluate the validity of the five latent variables: IRD, GRD, OI, OCB, and AR. The model demonstrated a good fit to the data, with the following indices: *χ*^2^/df = 2.183, RMSEA = 0.062, GFI = 0.902, AGFI = 0.973, IFI = 0.944, NNFI = 0.934, and CFI = 0.943. These results align with the established thresholds for acceptable model fit.

The composite reliability (CR) of each variable ranged from 0.820 to 0.928, all exceeding the critical threshold of 0.7, further confirming the scale’s reliability. The factor loading values for all items on their respective latent variables varied from 0.727 to 0.874 and passed the significance test at the 0.001 level, demonstrating solid convergent validity. The average variance extracted (AVE) analysis indicated that the AVE values for each variable ranged from 0.595 to 0.681, all surpassing the critical criterion of 0.5, further demonstrating satisfactory convergent validity. Additionally, the correlation coefficients between the variables were calculated and are presented in Table 2. The results indicated that the correlations among the variables ranged from 0.072 to 0.524, while the square roots of the AVE values for each variable ranged from 0.771 to 0.825. Significantly, the square root of each variable’s AVE exceeded the absolute values of its correlations with other variables, confirming that the variables were distinguishable, thereby demonstrating strong discriminant validity of the measurement scales.

**Table 2:** Descriptive statistics and correlation estimates were used.

Constructs	Mean	SD	IRD	GRD	OI	OCB	AR
IRD	4.99	0.84	0.777				
GRD	5.02	0.96	0.439**	0.816			
OI	4.88	1.03	−0.391**	−0.328**	0.825		
OCB	5.22	0.88	−0.449**	−0.524**	0.450**	0.775	
AR	4.75	0.86	−0.115	−0.072	0.221**	0.251**	0.771

Diagonal values represent the square root values of the AVE; **p < 0.01.

### 4.3 Hypothesis testing

#### 4.3.1 Direct effect test

Hierarchical regression analysis was performed using SPSS to assess the direct relationships among the variables. As summarized in Table 3, after controlling for gender, age, education level, and enterprise type, IRD and GRD were added to baseline Models M1 and M5, resulting in Models M2 and M6, respectively.

**Table 3:** Hierarchical regression results.

Variables	OCB				OI				
	M1	M2	M3	M4	M5	M6	M7	M8	M9
Gender	0.160**	0.120*	0.148**	0.124*	0.030	−0.015	−0.034	−0.038	−0.003
Age	0.034	0.014	0.021	0.011	0.034	0.013	0.004	−0.001	0.027
Education	0.003	0.016	−0.015	0.005	0.045	0.045	0.047	0.036	0.072
Enterprise	0.106	0.044	0.079	0.036	0.067	0.030	0.039	0.064	0.030
IRD		−0.213**		−0.146**		−0.257**	−0.245**	−0.312**	
GRD		−0.364**		−0.312**		−0.202**	−0.196**		−0.286**
OI			0.397**	0.259**					
AR							0.172**	0.166**	0.172**
IRD × AR								0.114*	
GRD × AR									0.127*
R^2^	0.041	0.270	0.197	0.326	0.010	0.151	0.180	0.161	0.147
△R^2^	0.041*	0.229**	0.156**	0.129**	0.010	0.141**	0.029**	0.151**	0.137**
F	3.192*	18.335**	14.694**	20.556**	0.752	8.858**	9.321**	8.129**	7.301**

*p < 0.05, **p < 0.01.

The results of Model M2 indicate that both IRD (*β* = −0.213, *p <* 0.01) and GRD (*β* = −0.364, *p <* 0.01) have significant adverse effects on OCB, supporting Hypotheses H1a and H1b, respectively. The subsequent analysis expanded Model M1 by incorporating OI as an independent variable (Model M3). The findings revealed that OI significantly influenced OCB (*β* = 0.397, *p <* 0.01), confirming H2. Further analysis using Model M6 showed that both the IRD (*β* = −0.257, *p <* 0.01) and GRD (*β* = −0.202, *p <* 0.01) negatively predicted OI.

#### 4.3.2 Mediation effect test

Following the mediation effect testing procedure, Model M4 was developed by incorporating the mediating variable (OI) into the baseline Model M2 to assess whether the mediating variable had a mediating effect. To mitigate the effects of multicollinearity, we centered both the independent and moderator variables prior to constructing their interaction term. The results from Model M4 showed that after including OI as the mediating variable, the relationships between IRD, GRD, and OCB remained significant (*β* = −0.146, *p <* 0.01; *β* = −0.312, *p <* 0.01), with coefficients lower than their corresponding direct effect path coefficients (*β* = −0.213, *p <* 0.01; *β* = −0.364, *p <* 0.01). This indicates that OI partially mediates the relationships between IRD, GRD, and OCB, thereby supporting H3a and H3b.

#### 4.3.3 Moderation effect test

To mitigate the risks of multicollinearity, the independent variables (IRD and GRD) and the moderating variable (AR) were mean-centered before creating the interaction terms. A hierarchical regression analysis was performed to investigate the moderating role of AR in the RD–OI relationship. The results in Table 3 (Models M8 and M9) reveal that the interaction terms between IRD and AR (*β* = 0.114, *p <* 0.05) and between GRD and AR (*β* = 0.127, *p <* 0.05) have a significant positive effect on OI. These findings indicate that AR mitigates the negative impact of IRD and GRD on OI, thus supporting H4a and H4b, respectively. Specifically, when employees attributed RD to internal factors (e.g., personal effort), the detrimental effect of deprivation on OI was lessened; however, external attributions (e.g., organizational inequity) intensified this negative relationship.

**Figure 2:**
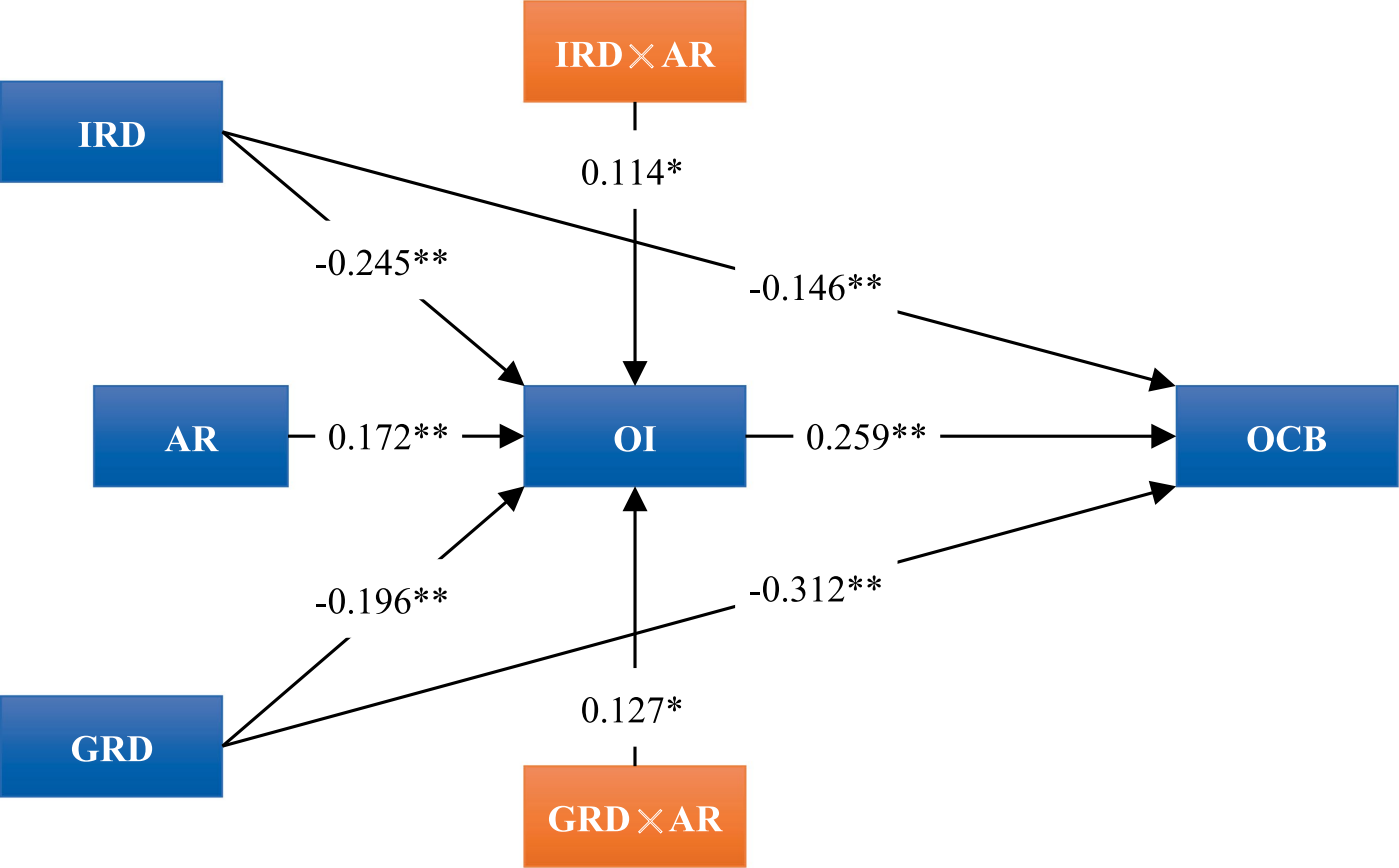
Depiction of the regression analysis outcomes (control variables are omitted). Values between paths represent the standardized path coefficients; *p < 0.05, **p < 0.01.

Figure 2 depicts the relationship model derived from the regression analysis conducted in this study. It clearly demonstrates the direct effects of IRD, GRD, and OI on OCB, as well as the direct effects of IRD and GRD, along with their interaction terms with AR, on OI.

#### 4.3.4 Test of the moderated mediation effect

To examine the moderated mediation effects, we employed the SPSS PROCESS macro (Model 7) to calculate the indirect effects of IRD and GRD on OCB through OI at different levels of AR. The results are shown in Table 4.

**Table 4:** Moderated mediation results.

Variables	Moderator	Moderated mediation effect			Index of moderated mediation		
		Effect	SE	95% confidence interval	Index	SE	95% confidence interval
IRD	High (M + 1SD)	−0.065	0.027	[−0.122, −0.014]	0.046	0.023	[0.007, 0.095]
	Low (M-1SD)	−0.144	0.035	[−0.216, −0.079]			
	Difference	0.079	0.039	[0.013, 0.164]			
GRD	High (M + 1SD)	−0.042	0.022	[−0.090, −0.005]	0.041	0.016	[0.011, 0.074]
	Low (M-1SD)	−0.112	0.026	[−0.168, −0.065]			
	Difference	0.071	0.028	[0.019, 0.128]			

For IRD, the indirect effect of OCB via OI was significant in both the high and low AR groups (mean ± one standard deviation). Specifically, the 95% confidence intervals for the indirect effects did not include zero in either group (high AR: [−0.122, −0.014]; low AR: [−0.216, −0.079]). Furthermore, the difference in indirect effects between the two groups was 0.079 (95% CI: [0.013, 0.164]), confirming the significant moderating role of the AR. A similar pattern emerged for GRD, where the indirect effects remained significant across AR levels (high AR: [−0.090, −0.005]; low AR: [−0.168, −0.065]), with a significant intergroup difference of 0.071 (95% CI: [0.019, 0.128]).

The moderated mediation indices (Index) were further examined. For IRD, the Index value was 0.046 (95% CI: [0.007, 0.095]), whereas for GRD, it was 0.041 (95% CI: [0.011, 0.074]). Both indices excluded zero from their confidence intervals, statistically supporting the presence of moderated mediation. These results indicate that AR significantly regulates the strength of the indirect pathways from RD to OCB via OI, thereby supporting H5a and H5b. Stronger tendencies toward internal attribution lessen the harmful effects of RD on OI, thereby weakening the negative mediation mechanism. In contrast, weaker internal attribution intensifies the damaging impact on OI, exacerbating its suppression of OCB.

**Figure 3:**
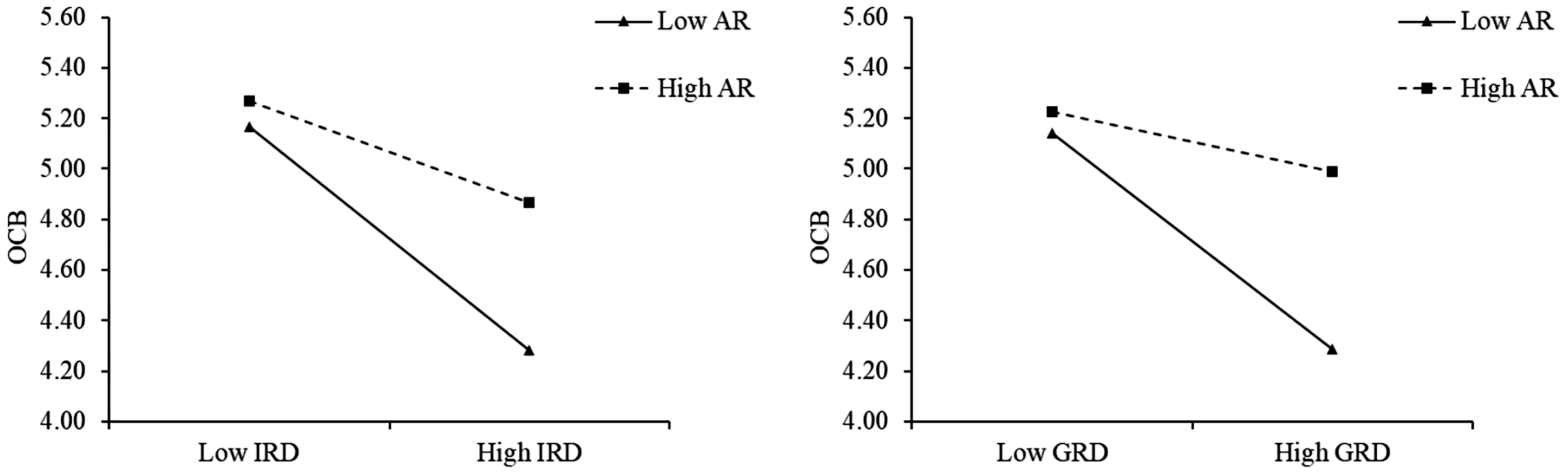
Moderated results of AR on the mediating effects.

To visually illustrate the aforementioned moderating effect, we categorized AR into high and low groups based on ±1 standard deviation and performed a simple slope analysis using the calculation results from the SPSS PROCESS macro. As depicted in Figure 3, for the low AR group, IRD and GRD exhibited a significantly negative predictive effect on OCB through OI; conversely, for the high AR group, the negative predictive effect of IRD and GRD was comparatively diminished.

## 5 Discussion

This study integrates Social Identity Theory and Attribution Theory to investigate how RD influences OCB among tourism employees, based on 305 valid survey responses. First, the findings of this study indicate that both IRD and GRD significantly and negatively affect employees’ OCB, but through distinct pathways: IRD directly undermines employees’ willingness to take on extra-role responsibilities by triggering unfavorable status perceptions through social comparison (Smith et al., 2012), whereas GRD reduces team collaboration effectiveness by reinforcing intergroup disadvantage perceptions (van Zomeren et al., 2008). These findings enhance our understanding of the varied impacts of RD and reveal a dual-path erosion mechanism in tourism enterprises: psychological imbalance stemming from individual emotional exhaustion and collective frustration that work together to suppress OCB (Cropanzano et al., 2003). Simultaneously, this study expands the theoretical boundaries of how negative psychological experiences influence OCB, addressing the research gap perpetuated by the predominant “positivity bias” in the literature. While traditional OCB studies emphasize the facilitative role of positive psychological constructs, such as psychological capital and thriving at work, the inhibitory mechanisms of negative affective states remain largely unexplored. By empirically validating the negative impact of RD on OCB, this study establishes the “eroding effect” of psychological imbalance on extra-role work contributions. Furthermore, by delineating the distinct mechanisms of individual and group RD, this study advances a multilevel understanding of the theoretical implications of deprivation while providing a novel framework for elucidating behavioral heterogeneity in high-emotional-labor contexts such as tourism.

Second, this study reveals that OI partially mediates the negative impact of IRD and GRD on OCB, confirming its role as a key transmission mechanism in the relationship between RD and OCB. This finding validates the core proposition of social identity theory: when employees experience organizational alienation due to deprivation, their weakened identification with organizational values reduces altruistic behavior and organizational loyalty through “identity decoupling” (Ashforth and Mael, 1989). While existing literature often treats OI as a stable antecedent or outcome variable (Riketta, 2005), this study positions it as a dynamic mediator between psychological perception and behavioral choice, emphasizing its “buffer” function in the context of RD. The discovery that weakened OI directly suppresses OCB supports Ashforth and Mael’s (1989) core hypothesis that “identity crises trigger behavioral withdrawal,” while also revealing tourism industry employees’ heightened sensitivity to fluctuations in OI due to occupational characteristics (e.g., uncontrollable service scenarios). Additionally, the finding that GRD weakens OI less severely than IRD aligns with Bagci et al.’s (2018) assertion that “group belonging mitigates self-threat,” highlighting the potential value of organizational culture in employee behavior intervention.

Third, the findings of this study indicate that AR modulates the transmission pathway of RD by reframing cognitive schemas, thereby establishing dynamic boundaries for behavioral choices. When employees attribute deprivation to internal factors, such as personal effort, the erosion of OI is reduced. In contrast, external attributions to systemic inequities worsen OI decline. Additionally, AR moderates the mediating role of OI in the RD-OCB connection, suggesting that cognitive reframing of deprivation experiences affects not only initial psychological responses but also recalibrates long-term behavioral patterns (Weiner, 1985). These findings provide a theoretical basis for psychological interventions to alleviate employees’ psychological imbalance, highlighting attribution guidance as a crucial management tool. Although Attribution Theory has long examined how causal explanations shape behavioral outcomes, its organizational applications remain concentrated in leadership and performance appraisal domains. By empirically demonstrating AR’s moderating role of AR in the RD → OI → OCB pathway, this study integrates attributional mechanisms into the dynamic transmission of deprivation, revealing the critical role of cognitive reframing in mitigating psychological imbalance. Specifically, internal attribution tendencies reduce identity erosion by activating self-regulatory processes, thereby diminishing the detrimental impact of RD on OI. This discovery empirically validates the applicability of Attribution Theory to service industry management. It offers novel theoretical pathways for investigating attributional preferences in cross-cultural settings, including societies shaped by Confucian ideals of self-reflection and social harmony.

Finally, this study’s theoretical contributions are particularly salient within the tourism industry, whose unique characteristics distinguish it from other sectors and amplify the psychological mechanisms investigated. Unlike the manufacturing or technology industries, tourism has its own industry-specific characteristics (Baum, 2018; Teng, 2019). Because tourism roles frequently require employees to perform tasks beyond their formal job descriptions to ensure customer satisfaction, OCB is not merely beneficial but essential for service quality and competitive advantages (Dai et al., 2018; Wu and Liao, 2016). Consequently, the erosion of OCB due to RD—a core finding of this study—poses a more immediate and significant threat to organizational effectiveness in tourism than in industries where discretionary behaviors are less critical to the core products or services. Therefore, by contextualizing the RD → OCB pathway within tourism, this study highlights how the industry’s inherent structural and social features create a fertile ground for such negative effects, underscoring the specific and critical value of these findings for both theory and service sector management.

## 6 Conclusion and limitations

### 6.1 Research conclusions

In conclusion, this study, by integrating Social Identity Theory and Attribution Theory, deeply investigates the mechanisms through which RD (including both individual and group levels) affects the OCB of tourism employees, and reveals the roles that OI and AR play in this complex relationship. The findings illuminate three key points: (1) RD exerts a significant inhibitory effect on OCB, meaning that both IRD and GRD significantly reduce employees’ engagement in OCB; (2) OI plays a vital partial mediating role in this relationship, whereby RD indirectly suppresses OCB by weakening employees’ OI; and (3) AR functions as a key moderator in this pathway, moderating the negative impact of RD on OI and the mediating role of OI in the connection between RD and OCB. When employees attribute deprivation to internal factors, such as personal effort, its erosive effect on OI is reduced.

The results also have practical implications. First, organizations should establish equitable and transparent resource allocation and communication mechanisms to mitigate the emergence of RD. To address the IRD, enterprises should conduct regular compensation satisfaction surveys and integrate performance feedback systems into promotion protocols. These measures can reduce employees’ perceived disadvantages from information asymmetries during horizontal comparisons. For the GRD, interdepartmental collaboration initiatives such as cross-functional training and job rotations can alleviate intergroup competition. Concurrently, organizations should develop shared interest mechanisms between the organization and groups to reinforce collective belonging.

Second, tourism enterprises should reinforce the buffering effect of OI through cultural immersion and identity enhancement. For instance, integrating corporate value narratives into onboarding programs and organizing ritualized activities, such as annual recognition ceremonies, can deepen these emotional bonds. Additionally, granting participatory decision-making rights to frontline employees fosters psychological empowerment, enabling them to internalize organizational goals as personal values.

Third, attributional guidance strategies should be designed to reframe employees’ cognitive interpretations of their deprivation. Managers can introduce scenario-based training sessions that enable employees to differentiate between internal (e.g., lack of effort) and external factors (e.g., structural inefficiencies). By reframing setbacks as opportunities for growth, such approaches stimulate adaptive coping behaviors. Additionally, managers may implement attribution guidance initiatives, such as organizing workshops during off-peak seasons that reframe RD through activities focused on collective achievement, such as team-building exercises.

### 6.2 Limitations and future directions

Despite the theoretical and methodological rigor of this study, several limitations warrant acknowledgment and present opportunities for future studies. First, the sample primarily consisted of employees from traditional tourism sectors (e.g., hotels and scenic areas), potentially underrepresenting emerging digital platforms such as online travel agencies and shared accommodation providers, potentially limiting generalizability to emerging formats such as online travel agencies. These entities often exhibit distinct organizational dynamics and employee experiences owing to technology-mediated workflows and decentralized management structures. Future studies should include data from multiple types of tourism enterprises to enhance research generalizability.

Second, the cross-sectional design and reliance on self-reported data inherently limit causal inferences between RD and OCB. While Harman’s single-factor test mitigates concerns about common method bias, longitudinal or experimental designs can better capture temporal dynamics. For instance, tracking employees’ psychological states and behavioral patterns during organizational restructuring initiatives could elucidate the causal mechanisms and temporal lags in the relationships between RDs and OCBs.

Third, the theoretical model focuses on the moderating role of internal factors such as AR, potentially overlooking external attributions (e.g., market competition) and psychological contracts. These factors may systematically shape how perceptions of deprivation translate into behavioral outcomes. Incorporating multilevel organizational variables can refine boundary conditions and strengthen practical relevance.

Finally, the cultural specificity of attribution patterns remains to be explored. Given the Confucian values emphasizing self-effacement and collective harmony in Chinese contexts, cross-cultural comparisons between individualistic (e.g., North American) and collectivistic (e.g., East Asian) societies could clarify how cultural norms interact with AR to influence behavioral responses in different cultural contexts.

Future studies should utilize multisource data triangulation to reduce the inherent methodological common variance of self-report methods. Complementary mixed-method designs, including longitudinal interviews and experience sampling methodologies, can further elucidate the contextual mechanisms underlying quantitative patterns. Additionally, investigating boundary conditions, such as equity-enhancing HR practices or evidence-based resilience interventions, may yield actionable frameworks for tourism enterprises operating in hyper-competitive and uncertain market environments.

## Data Availability

The original contributions presented in the study are included in the article/supplementary material, further inquiries can be directed to the corresponding author.

## References

[ref1] Anitha E. G. N. Suganthi L. Rajesh J. I. Sumathi G. N. (2024). Emotional contagion—as a moderator in personality and organizational citizenship behavior relationship. Bus. Perspect. Res. 12, 277–295. doi: 10.1177/22785337221148857

[ref2] Ashforth B. E. Mael F. (1989). Social identity theory and the organization. Acad. Manag. Rev. 14, 20–39. doi: 10.2307/258189

[ref3] Bagci S. C. Turnuklu A. Bekmezci E. (2018). The buffering role of in-group identification and intergroup contact on the association between perceived discrimination and mental health. J. Community Appl. Soc. Psychol. 28, 293–305. doi: 10.1002/casp.2357

[ref4] Bagozzi R. P. (1992). The self-regulation of attitudes, intentions, and behavior. Soc. Psychol. Q. 55, 178–204. doi: 10.2307/2786945

[ref5] Baum T. (2018). Sustainable human resource management as a driver in tourism policy and planning: a serious sin of omission? J. Sustain. Tour. 26, 873–889. doi: 10.1080/09669582.2017.1423318

[ref6] Bergami M. Bagozzi R. P. (2000). Self-categorization, affective commitment and group self-esteem as distinct aspects of social identity in the organization. Br. J. Soc. Psychol. 39, 555–577. doi: 10.1348/014466600164633, PMID: 11190685

[ref7] Bolino M. C. Klotz A. C. Turnley W. H. Harvey J. (2013). Exploring the dark side of organizational citizenship behavior. J. Organ. Behav. 34, 542–559. doi: 10.1002/job.1847

[ref8] Bugental D. B. (1987). Attributions as moderator variables within social interactional systems. J. Soc. Clin. Psychol. 5, 469–484. doi: 10.1521/jscp.1987.5.4.469

[ref9] Carver C. S. Scheier M. F. (1998). On the self-regulation of behavior. New York, NY: Cambridge University Press.

[ref10] Chaudhary R. Srivastava S. Singh L. B. (2024). Does workplace ostracism lead to knowledge hiding? Modeling workplace withdrawal as a mediator and authentic leadership as a moderator. Int. J. Hum. Resour. Manag. 35, 3593–3636. doi: 10.1080/09585192.2024.2424289

[ref11] Cheney G. (1983). On the various and changing meanings of organizational membership: a field study of organizational identification. Commun. Monogr. 50, 342–362. doi: 10.1080/03637758309390174

[ref12] Chi N. W. Han T. S. (2008). Exploring the linkages between formal ownership and psychological ownership for the organization: the mediating role of organizational justice. J. Occup. Organ. Psychol. 81, 691–711. doi: 10.1348/096317907X262314

[ref13] Choi J.-G. Woods R. H. Murrmann S. K. (2000). International labor markets and the migration of labor forces as an alternative solution for labor shortages in the hospitality industry. Int. J. Contemp. Hospit. Manag. 12, 61–67. doi: 10.1108/09596110010305154

[ref14] Cordeiro J. P. Pitacho L. Lima D. (2024). Organizational citizenship behaviors in the Portuguese hospitality industry: a study on sociodemographic and professional variables. Soc. Sci. 13:315. doi: 10.3390/socsci13060315

[ref15] Costa C. Bakas F. E. Breda Z. Durão M. Carvalho I. Caçador S. (2017). Gender, flexibility and the ‘ideal tourism worker’. Ann. Tour. Res. 64, 64–75. doi: 10.1016/j.annals.2017.03.002

[ref16] Cropanzano R. Mitchell M. S. (2005). Social exchange theory: an interdisciplinary review. J. Manage. 31, 874–900. doi: 10.1177/0149206305279602

[ref17] Cropanzano R. Rupp D. E. Byrne Z. S. (2003). The relationship of emotional exhaustion to work attitudes, job performance, and organizational citizenship behaviors. J. Appl. Psychol. 88, 160–169. doi: 10.1037/0021-9010.88.1.160, PMID: 12675403

[ref18] Dai Y. D. Chen K. Y. Zhuang W. L. (2016). Moderating effect of work–family conflict on the relationship between leader–member exchange and relative deprivation: links to behavioral outcomes. Tour. Manag. 54, 369–382. doi: 10.1016/j.tourman.2015.12.005

[ref19] Dai Y. D. Hou Y. H. Chen K. Y. Zhuang W. L. (2018). To help or not to help: antecedents of hotel employees’ organizational citizenship behavior. Int. J. Contemp. Hospit. Manag. 30, 1293–1313. doi: 10.1108/IJCHM-03-2016-0160

[ref20] Dai Y. Tang Y. M. Chen W. Hou J. (2022). How organizational trust impacts organizational citizenship behavior: organizational identification and employee loyalty as mediators. Front. Psychol. 13:996962. doi: 10.3389/fpsyg.2022.996962, PMID: 36457918 PMC9706095

[ref21] Divya S. Christopher B. P. (2024). A decadal review of organizational identification: insights from bibliometric analysis and content analysis. Humanit. Soc. Sci. Commun. 11, 1–21. doi: 10.1057/s41599-024-03990-7

[ref22] Dukerich J. M. Golden B. R. Shortell S. M. (2002). Beauty is in the eye of the beholder: the impact of organizational identification, identity, and image on the cooperative behaviors of physicians. Adm. Sci. Q. 47, 507–533. doi: 10.2307/3094849

[ref23] Dweck C. S. (2013). Self-theories: Their role in motivation, personality, and development. London: Psychology Press. 2130257

[ref24] Eberly M. B. Holley E. C. Johnson M. D. Mitchell T. R. (2011). Beyond internal and external: a dyadic theory of relational attributions. Acad. Manag. Rev. 36, 731–753. doi: 10.5465/AMR.2011.65554734

[ref25] Eisenberger R. Armeli S. Rexwinkel B. Lynch P. D. Rhoades L. (2001). Reciprocation of perceived organizational support. J. Appl. Psychol. 86, 42–51. doi: 10.1037/0021-9010.86.1.42, PMID: 11302232

[ref26] Faul F. Erdfelder E. Lang A.-G. Buchner A. (2007). G*Power 3: a flexible statistical power analysis program for the social, behavioral, and biomedical sciences. Behav. Res. Methods 39, 175–191. doi: 10.3758/BF03193146, PMID: 17695343

[ref27] Foster C. A. Rusbult C. E. (1999). Injustice and powerseeking. Personal. Soc. Psychol. Bull. 25, 834–849. doi: 10.1177/0146167299025007006

[ref28] Golverdi M. Sharifirad M. S. Rastegar R. (2024). Mapping organizational justice in tourism, hospitality, and events literature: an in-depth scoping review. J. Hosp. Tour. Manag. 60, 22–32. doi: 10.1016/j.jhtm.2024.06.004

[ref29] Grant A. M. Parker S. K. (2009). Redesigning work design theories: the rise of relational and proactive perspectives. Acad. Manage. Ann. 3, 317–375. doi: 10.5465/19416520903047327

[ref30] Guimond S. Dubé-Simard L. (1983). Relative deprivation theory and the Quebec nationalist movement: the cognition–emotion distinction and the personal–group deprivation issue. J. Pers. Soc. Psychol. 44, 526–535. doi: 10.1037/0022-3514.44.3.526

[ref31] Gupta V. Mittal S. Ilavarasan P. V. Budhwar P. (2024). Pay-for-performance, procedural justice, OCB and job performance: a sequential mediation model. Pers. Rev. 53, 136–154. doi: 10.1108/PR-11-2021-0782

[ref32] Hsu C. H. C. Chen N. (2019). Resident attribution and tourist stereotypes. J. Hosp. Tour. Res. 43, 489–516. doi: 10.1177/1096348018823903

[ref33] Jamal A. Begum S. Alam H. Hussain T. (2023). Impact of perceived organizational support on innovative work behavior and burnout in teachers: thriving at work as the mediator (2020). J. Educ. Educ. Dev. 10, 288–307. doi: 10.22555/joeed.v10i2.815

[ref34] Jolliffe L. Farnsworth R. (2003). Seasonality in tourism employment: human resource challenges. Int. J. Contemp. Hospit. Manag. 15, 312–316. doi: 10.1108/09596110310488140

[ref35] Junça-Silva A. Lopes E. (2023). Testing the affective events theory in hospitality management: a multi-sample approach. Sustainability 15:7168. doi: 10.3390/su15097168

[ref36] Kao J. C. Cho C. C. Kao R. H. (2023). Perceived organizational support and organizational citizenship behavior—a study of the moderating effect of volunteer participation motivation, and cross-level effect of transformational leadership and organizational climate. Front. Psychol. 14:1082130. doi: 10.3389/fpsyg.2023.1082130, PMID: 36844327 PMC9947714

[ref37] Karacay G. Rofcanin Y. Kabasakal H. (2023). Relative leader–member exchange perceptions and employee outcomes in service sector: the role of self-construal in feeling relative deprivation. Int. J. Hum. Resour. Manag. 34, 1808–1851. doi: 10.1080/09585192.2022.2037097

[ref38] Karatepe O. M. (2013). High-performance work practices and hotel employee performance: the mediation of work engagement. Int. J. Hosp. Manag. 32, 132–140. doi: 10.1016/j.ijhm.2012.05.003

[ref39] Kim H. Callan M. J. Gheorghiu A. I. Skylark W. J. (2018). Social comparison processes in the experience of personal relative deprivation. J. Appl. Soc. Psychol. 48, 519–532. doi: 10.1111/jasp.12531

[ref40] Kreiner G. E. Ashforth B. E. (2004). Evidence toward an expanded model of organizational identification. J. Organ. Behav. 25, 1–27. doi: 10.1002/job.234

[ref41] Kurtessis J. N. Eisenberger R. Ford M. T. Buffardi L. C. Stewart K. A. Adis C. S. (2017). Perceived organizational support: a meta-analytic evaluation of organizational support theory. J. Manage. 43, 1854–1884. doi: 10.1177/0149206315575554

[ref42] Lee M. C. C. Lin M. H. Srinivasan P. M. Carr S. C. (2024). Transformational leadership and organizational citizenship behavior: new mediating roles for trustworthiness and trust in team leaders. Curr. Psychol. 43, 9567–9582. doi: 10.1007/s12144-023-05095-x

[ref43] Lee L. Madera J. M. (2019). A systematic literature review of emotional labor research from the hospitality and tourism literature. Int. J. Contemp. Hospit. Manag. 31, 2808–2826. doi: 10.1108/IJCHM-05-2018-0395

[ref44] Lee E.-S. Park T.-Y. Koo B. (2015). Identifying organizational identification as a basis for attitudes and behaviors: a meta-analytic review. Psychol. Bull. 141, 1049–1080. doi: 10.1037/bul0000012, PMID: 25984729

[ref45] Lee J. Shin H. (2024). Effects of inclusive leadership on the diversity climate and change-oriented organizational citizenship behavior. Behav. Sci., 14, 491, doi: 10.3390/bs14060491, PMID: 38920823 PMC11200651

[ref46] Li Z. Liang W. Bao Y. Zhang R. (2022). The role of relative deprivation and attribution style in the relationship between organizational fairness and employees’ service innovation behavior. Behavioral Sci. 12:506. doi: 10.3390/bs12120506, PMID: 36546989 PMC9774929

[ref47] Liang Z. Geng M. Yue J. (2019). Development of the group relative deprivation of enterprise employees questionnaire. J. Mudanjiang Normal Univ., 30–38. doi: 10.13815/j.cnki.jmtc(pss).2019.04.004

[ref48] Liu Y. Yu S. Ding H. (2024). Strengths mindset and organizational citizenship behavior: the roles of thriving at work and guanxi closeness. Curr. Psychol. 43, 28797–28807. doi: 10.1007/s12144-024-06531-2

[ref49] Luthans F. Avolio B. J. Avey J. B. Norman S. M. (2007). Positive psychological capital: measurement and relationship with performance and satisfaction. Pers. Psychol. 60, 541–572. doi: 10.1111/j.1744-6570.2007.00083.x

[ref50] Mael F. Ashforth B. E. (1992). Alumni and their alma mater: a partial test of the reformulated model of organizational identification. J. Organ. Behav. 13, 103–123. doi: 10.1002/job.4030130202

[ref51] Marescaux E. De Winne S. Rofcanin Y. (2021). Co-worker reactions to i-deals through the lens of social comparison: the role of fairness and emotions. Hum. Relat. 74, 329–353. doi: 10.1177/0018726719884103

[ref52] McManus H. Dundon T. Lavelle J. (2025). “Workin for a livin”: mediating the role of perceived support, work engagement, and organizational citizenship behavior in the hospitality sector. Int. J. Hospit. Manag. 126:103983. doi: 10.1016/j.ijhm.2024.103983

[ref53] Melkonian T. Monin P. Noorderhaven N. G. (2011). Distributive justice, procedural justice, exemplarity, and employees' willingness to cooperate in M&a integration processes: an analysis of the air France-KLM merger. Hum. Resour. Manag. 50, 809–837. doi: 10.1002/hrm.20456

[ref54] Meyer J. P. Allen N. J. (1991). A three-component conceptualization of organizational commitment. Hum. Resour. Manag. Rev. 1, 61–89. doi: 10.1016/1053-4822(91)90011-Z

[ref55] Ohno H. Lee K. T. Maeno T. (2023). Feelings of personal relative deprivation and subjective well-being in Japan. Behav. Sci. 13:158. doi: 10.3390/bs13020158, PMID: 36829387 PMC9952549

[ref56] Organ D. W. (1988). Organizational citizenship behavior: The good soldier syndrome. London: Lexington Books.

[ref57] Osborne D. Huo Y. J. Smith H. J. (2015). Organizational respect dampens the impact of group-based relative deprivation on willingness to protest pay cuts. Br. J. Soc. Psychol. 54, 159–175. doi: 10.1111/bjso.12069, PMID: 24690102

[ref58] Pan J. Yang Z. (2023). Knowledge mapping of relative deprivation theory and its applicability in tourism research. Humanit. Soc. Sci. Commun. 10:68. doi: 10.1057/s41599-023-01520-5

[ref59] Pettigrew T. F. Christ O. Wagner U. Meertens R. W. van Dick R. Zick A. (2008). Relative deprivation and intergroup prejudice. J. Soc. Issues 64, 385–401. doi: 10.1111/j.1540-4560.2008.00567.x

[ref60] Podsakoff P. M. MacKenzie S. B. Lee J. Y. Podsakoff N. P. (2003). Common method biases in behavioral research: a critical review of the literature and recommended remedies. J. Appl. Psychol. 88, 879–903. doi: 10.1037/0021-9010.88.5.879, PMID: 14516251

[ref61] Podsakoff P. M. MacKenzie S. B. Moorman R. H. Fetter R. (1990). Transformational leader behaviors and their effects on followers’ trust in leader, satisfaction, and organizational citizenship behaviors. Leadersh. Q. 1, 107–142. doi: 10.1016/1048-9843(90)90009-7

[ref62] Podsakoff P. M. MacKenzie S. B. Paine J. B. Bachrach D. G. (2000). Organizational citizenship behaviors: a critical review of the theoretical and empirical literature and suggestions for future research. J. Manage. 26, 513–563. doi: 10.1177/014920630002600307

[ref63] Power S. A. Madsen T. Morton T. A. (2020). Relative deprivation and revolt: current and future directions. Curr. Opin. Psychol. 35, 119–124. doi: 10.1016/j.copsyc.2020.06.010, PMID: 32674060

[ref65] Riketta M. (2005). Organizational identification: a meta-analysis. J. Vocat. Behav. 66, 358–384. doi: 10.1016/j.jvb.2004.05.005

[ref66] Schreurs B. Hamstra M. R. W. Jawahar I. M. Akkermans J. (2021). Perceived overqualification and counterproductive work behavior: testing the mediating role of relative deprivation and the moderating role of ambition. Pers. Rev. 50, 1038–1055. doi: 10.1108/PR-05-2019-0237

[ref67] Smith H. J. Ortiz D. J. (2002). “Is it just me?:the different consequences of personal and group relative deprivation” in Relative deprivation: Specification, development, and integration. eds. Walker I. Smith H. J. (New York, NY: Cambridge University Press), 91–116.

[ref68] Smith H. J. Pettigrew T. F. (2015). Advances in relative deprivation theory and research. Soc. Justice Res 28, 1–6. doi: 10.1007/s11211-014-0231-5

[ref69] Smith H. J. Pettigrew T. F. Pippin G. M. Bialosiewicz S. (2012). Relative deprivation: a theoretical and meta-analytic review. Personal. Soc. Psychol. Rev. 16, 203–232. doi: 10.1177/1088868311430825, PMID: 22194251

[ref70] Stouffer S. A. Suchman E. A. DeVinney L. C. Star S. A. Williams R. M. Jr. (1949). The American soldier: Adjustment during Army life. New Jersey: Princeton University Press.

[ref71] Su L. Gong Q. Huang Y. (2020). How do destination social responsibility strategies affect tourists’ intention to visit? An attribution theory perspective. J. Retail. Consum. Serv. 54:102023. doi: 10.1016/j.jretconser.2019.102023

[ref72] Su S. Zhang J. Xia L. X. (2023). The relationship between group relative deprivation and aggressive collective action online toward deprivation-related provocateurs within the group: the mediating role of hostile feelings. Curr. Psychol. 42, 25246–25256. doi: 10.1007/s12144-022-03530-z, PMID: 35990206 PMC9382011

[ref73] Sumardjo M. Supriadi Y. N. (2023). Perceived organizational commitment mediates the effect of perceived organizational support and organizational culture on organizational citizenship behavior. Quality-Access to Success 24, 376–384. doi: 10.47750/QAS/24.192.45

[ref74] Teng H. Y. (2019). Job crafting and customer service behaviors in the hospitality industry: mediating effect of job passion. Int. J. Hosp. Manag. 81, 34–42. doi: 10.1016/j.ijhm.2019.03.013

[ref75] Teng C. C. Lu A. C. C. Huang Z. Y. Fang C. H. (2020). Ethical work climate, organizational identification, leader-member-exchange (LMX) and organizational citizenship behavior (OCB): a study of three star hotels in Taiwan. Int. J. Contemp. Hospit. Manag. 32, 212–229. doi: 10.1108/IJCHM-07-2018-0563

[ref76] Tiamiyu T. Quoquab F. Mohammad J. (2020). To switch or not to switch: the role of tourists’ psychological engagement in the context of Airbnb Malaysia. Int. J. Tour. Cities 6, 175–196. doi: 10.1108/IJTC-09-2019-0158

[ref77] Tourky M. Osman S. Harvey W. S. (2023). Aligning employee and organizational values to build organizational reputation. Asian Bus. Manag. 22, 1618–1648. doi: 10.1057/s41291-023-00223-8

[ref78] Tropp L. R. Wright S. C. (1999). Ingroup identification and relative deprivation: an examination across multiple social comparisons. Eur. J. Soc. Psychol. 29, 707–724. doi: 10.1002/(SICI)1099-0992(199908/09)29

[ref79] Tuan L. T. Rowley C. Masli E. Le V. Nhi L. T. P. (2021). Nurturing service-oriented organizational citizenship behavior among tourism employees through leader humility. J. Hosp. Tour. Manag. 46, 456–467. doi: 10.1016/j.jhtm.2021.02.001

[ref80] Turnipseed D. L. (2002). Are good soldiers good?: exploring the link between organizational citizenship behavior and personal ethics. J. Bus. Res. 55, 1–15. doi: 10.1016/S0148-2963(01)00217-X

[ref81] Van Knippenberg D. Sleebos E. (2006). Organizational identification versus organizational commitment: self-definition, social exchange, and job attitudes. J. Organ. Behav. 27, 571–584. doi: 10.1002/job.359

[ref82] van Zomeren M. Postmes T. Spears R. (2008). Toward an integrative social identity model of collective action: a quantitative research synthesis of three socio-psychological perspectives. Psychol. Bull. 134, 504–535. doi: 10.1037/0033-2909.134.4.504, PMID: 18605818

[ref83] Walker I. Smith H. J. (2002). Relative deprivation: Specification, development, and integration. New York, NY: Cambridge University Press.

[ref84] Walumbwa F. O. Avolio B. J. Gardner W. L. Wernsing T. S. Peterson S. J. (2008). Authentic leadership: development and validation of a theory-based measure. J. Manage. 34, 89–126. doi: 10.1177/0149206307308913

[ref85] Wang P. Qin C. Liu S. (2023). Relative deprivation, perceived status conflict and innovative behavior of outsourced employees: multiple moderating effects of dual organizational support. Pers. Rev. 52, 1071–1093. doi: 10.1108/PR-04-2021-0280

[ref86] Weiner B. (1985). An attributional theory of achievement motivation and emotion. Psychol. Rev. 92, 548–573. doi: 10.1037/0033-295X.92.4.548, PMID: 3903815

[ref87] Wright S. C. Taylor D. M. Moghaddam F. M. (1990). Responding to membership in a disadvantaged group: from acceptance to collective protest. J. Pers. Soc. Psychol. 58, 994–1003. doi: 10.1037/0022-3514.58.6.994

[ref88] Wu P. H. Liao J. F. (2016). Service-oriented organizational citizenship behavior, perceived service quality and customer satisfaction in hospitality industry. J. Appl. Sci. 16, 18–24. doi: 10.3923/jas.2016.18.24

[ref89] Yu S. Liu S. Gong X. Liu C. E. Cai W. (2025). Online platform algorithmic control and gig workers’ turnover intention in China: the mediating role of relative deprivation. J. Manage. Sci. Eng. 10, 37–53. doi: 10.1016/j.jmse.2024.08.004

[ref90] Zhang D. Ma Q. Zhao Z. (2020). A study on the cause and effect variables of relative deprivation of rural tourism residents: an individual-based psychological perspective. Hum. Geogr. 35, 32–39. doi: 10.13959/j.issn.1003-2398.2020.04.005

[ref91] Zuo B. Ye H. Wen F. Ke W. Xiao H. Wang J. (2023). Effect of attribution on the emotions and behavioral intentions of third-party observers toward intergroup discriminators during the COVID-19 pandemic. Group Process. Intergroup Relat. 26, 431–452. doi: 10.1177/13684302211062367, PMID: 36816350 PMC9922659

