# Supplementary material for: From psychological imbalance to behavioral withdrawal: unraveling the impact of relative deprivation on organizational citizenship behavior in tourism enterprises
Source: Front Psychol. 2025 Sep 19;16:1619960. doi: 10.3389/fpsyg.2025.1619960 (PMC12490995; doi:10.3389/fpsyg.2025.1619960)
Supplement: Supplementary file 1 [file Data_Sheet_1.pdf]

Appendix A. Measurement items.

**Variables**	
Relative deprivation	
Individual relative deprivation	IRD1: I believe my circumstances are worse than others.<br>IRD2: I believe I am being mistreated.<br>IRD3: Compared to those around me, my working conditions and treatment are significantly disadvantaged.
Group relative deprivation	GRD1: Compared to other groups, my group's status is lower.<br>GRD2: Our group's treatment is inferior to that of other groups.<br>GRD3: Our group's situation is worse than that of other groups.<br>GRD4: Good career prospects are not available to our type of group.
Organizational identification	
OI1: When someone criticizes my job, it feels like a personal attack.<br>OI2: I am pretty interested in how others view my workplace.<br>OI3: When talking about my workplace, I usually say "we" rather than "they."<br>OI4: The success of my workplace reflects my success.<br>OI5: When someone compliments my workplace, it feels like a personal compliment.<br>OI6: If the media were to criticize my workplace, I would feel embarrassed.	
Organizational citizenship behavior	
OCB1: I adhere to the workplace rules and regulations, even when no one supervises me.<br>OCB2: I try to avoid creating problems for my colleagues.<br>OCB3: I am always prepared to assist my coworkers.<br>OCB4: I actively work to enhance my organization's reputation, even when it goes beyond the basic requirements.	
Attribution of responsibility	
AR1: My disadvantaged situation stems from my lack of ability.<br>AR2: My disadvantaged situation stems from my lack of effort.<br>AR3: My disadvantaged situation stems from my low level of education.<br>AR4: My disadvantaged situation stems from my personality issues.	

Appendix B. List of constructs and items with means, standard deviations skew, and kurtosis.

Construct	Item	Mean	STD	Skew	Kurtosis
Individual relative deprivation	IRD1	4.97	0.971	-0.223	-0.759
	IRD2	4.97	1.038	-0.129	-0.823
	IRD3	5.03	0.928	-0.127	-0.912
Group relative deprivation	GRD1	5.00	1.124	0.098	-0.936
	GRD2	4.97	1.150	0.169	-0.885
	GRD3	5.02	1.144	0.001	-0.819
	GRD4	5.09	1.022	0.367	-0.777
Organizational identification	OI1	4.88	1.247	-0.414	-0.172
	OI2	4.91	1.243	-0.117	-0.489
	OI3	4.97	1.296	-0.064	-0.791
	OI4	4.83	1.158	-0.475	-0.039
	OI5	4.79	1.055	-0.130	-0.316
	OI6	4.92	1.199	-0.325	-0.448
Organizational citizenship behavior	OCB1	5.25	1.044	-0.216	0.092
	OCB2	5.22	1.023	-0.173	-0.198
	OCB3	5.16	1.115	-0.213	-0.425
	OCB4	5.25	1.044	-0.199	-0.231

Attribution of responsibility	AR1	4.66	1.046	-0.115	-0.389
	AR2	4.82	1.082	-0.068	-0.240
	AR3	4.76	1.003	0.179	-0.462
	AR4	4.75	1.012	0.074	-0.543

Appendix C. Socio-demographics of respondents (*n* = 305).

Demographics	Category	Frequency	Percent (%)
Gender	Male	143	46.89
	Female	162	53.11
Age	18 to 20	15	4.92
	21 to 30	137	44.92
	31 to 40	104	34.10
	41 or older	49	16.07
Education	Middle school or under	91	29.84
	High school	109	35.74
	University or college	99	32.46
	Master or doctor	6	1.97
Enterprise	travel agency	30	9.84
	tourist shop	39	12.79
	scenic area	75	24.59
	hotel	145	47.54
	others	16	5.25

Appendix D. Cross-loadings.

	IRD	GRD	OI	OCB	AR
IRD1	**0.768**	0.180	-0.242	-0.185	0.001
IRD2	**0.854**	0.088	-0.096	-0.113	-0.058
IRD3	**0.823**	0.207	-0.124	-0.130	-0.043
GRD1	0.108	**0.791**	-0.141	-0.269	0.022
GRD2	0.198	**0.825**	-0.066	-0.167	-0.030
GRD3	0.149	**0.838**	-0.118	-0.077	0.021
GRD4	0.061	**0.852**	-0.164	-0.200	-0.053
OI1	-0.137	-0.154	**0.823**	0.191	0.055
OI2	-0.025	-0.115	**0.805**	0.126	0.114
OI3	-0.114	-0.042	**0.849**	0.088	0.035
OI4	-0.073	-0.155	**0.790**	0.195	0.063
OI5	-0.154	-0.111	**0.817**	0.163	0.104
OI6	-0.105	-0.034	**0.886**	0.082	0.088
OCB1	-0.101	-0.106	0.193	**0.789**	0.050
OCB2	-0.124	-0.297	0.171	**0.734**	0.097

OCB3	-0.112	-0.144	0.210	**0.800**	0.102
OCB4	-0.147	-0.193	0.130	**0.802**	0.100
AR1	-0.047	-0.055	0.075	0.051	**0.809**
AR2	-0.076	-0.017	0.139	0.066	**0.789**
AR3	0.020	0.054	0.051	0.059	**0.850**
AR4	-0.002	-0.020	0.068	0.117	**0.852**
